# Unexpected Causes and Complications of ST-Segment Myocardial Infarction That Highlight the Importance and Limitations of Point-of-Care Ultrasound in the Emergency Department

**DOI:** 10.7759/cureus.35754

**Published:** 2023-03-04

**Authors:** Jordan Johnson, Daniel Romine, Matthew Flannigan, Joshua C Reynolds

**Affiliations:** 1 Emergency Medicine, Michigan State University College of Human Medicine, Grand Rapids, USA; 2 Emergency Medicine, Corewell Health West, Grand Rapids, USA

**Keywords:** myocardial infarction, valvular endocarditis, mitral valve insufficiency, echocardiogram (echo), point-of-care-ultrasound

## Abstract

Point-of-care transthoracic echocardiography is a valuable tool for Emergency Physicians evaluating a patient in shock. We describe a case report of ST-segment myocardial infarction complicated by cardiogenic shock and acute severe mitral valve regurgitation that was immediately identified by the Emergency Physician. However, subsequent testing revealed an unexpected unifying diagnosis. The diagnostic sequence in this case highlights the benefits and limitations of point-of-care ultrasound in the Emergency Department and reinforces its role to address discrete clinically relevant questions.

## Introduction

Cardiogenic shock complicates 7-10% of cases of acute myocardial infarction [1]. Papillary muscle rupture with acute valvular regurgitation is an uncommon cause of cardiogenic shock that carries a high mortality rate [2]. Emergency physicians (EPs) can identify evidence of papillary muscle rupture on point-of-care transthoracic echocardiography (POCTE) in patients with cardiogenic shock [3,4]. Early diagnosis of this emergent condition is useful to direct subsequent care. Here we present an unusual case of an elderly male with ST-segment elevation myocardial infarction (STEMI) complicated by cardiogenic shock who had an acute coronary occlusion, papillary muscle rupture with acute severe mitral regurgitation, and an unexpected unifying diagnosis. POCTE performed by the EP identified the severe valvular insufficiency and subsequent comprehensive transesophageal echocardiography (TEE) established the unifying diagnosis.

## Case presentation

**Figure 1:**
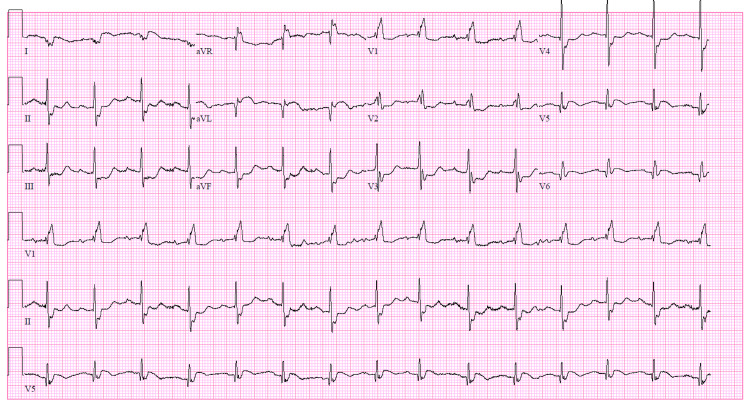
Initial electrocardiogram Electrocardiogram showing sinus rhythm at 88 beats per minute with a first-degree AV block, right bundle branch block, ST segment elevation in the lateral leads, and aVR with reciprocal ST segment depression in the inferior and septal leads.

An 80-year-old male with underlying hypertension, hyperlipidemia, paroxysmal atrial fibrillation (not anticoagulated), tobacco abuse, prostate cancer, and alcohol use disorder presented to an urban emergency department (ED) with three days of intermittent squeezing chest pain that became severe and unrelenting several hours prior to arrival. Upon initial evaluation, he was hypotensive (56/36 mmHg), but awake, not tachycardic, and oxygenating normally. His skin was pale, cool, and diaphoretic. His lungs had posterior inspiratory crackles but his lower extremities were not edematous. Electrocardiogram (ECG) demonstrated sinus rhythm with ST-segment elevation in the lateral leads and aVR with reciprocal ST-segment depression in the inferior and septal leads (Figure 1). Given the clinical impression of STEMI with associated cardiogenic shock, the patient received aspirin, heparin, norepinephrine, and dobutamine infusions, and urgent cardiology consultation for coronary angiography and revascularization as indicated.

**Figure 2:**
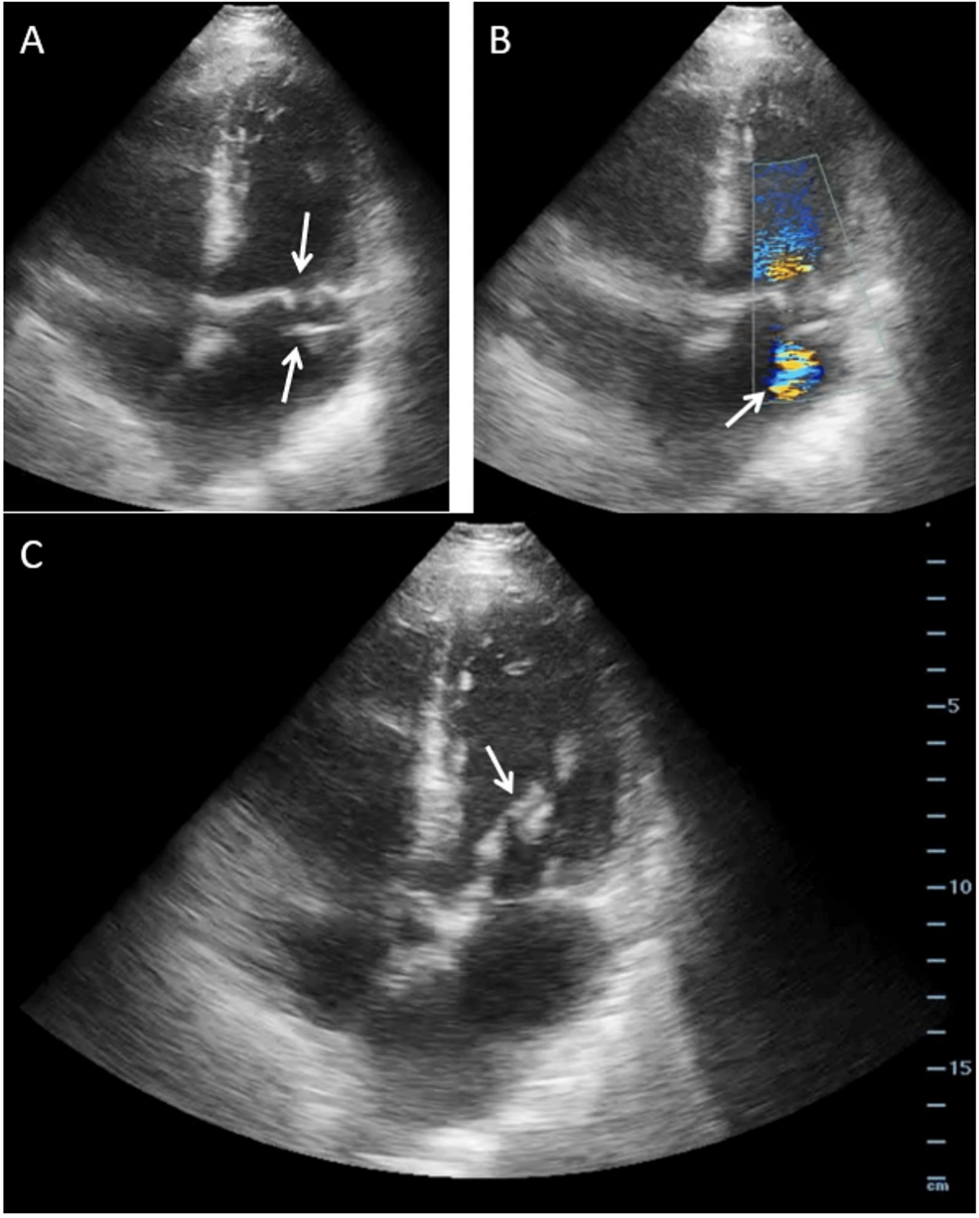
Bedside transthoracic echocardiography Bedside transthoracic echocardiography (apical 4-chamber view) performed by the emergency department (ED) resident physician demonstrating (A) retrograde movement of both mitral valve leaflets into the left atrium (downward arrow) with mass adherent to the anterior leaflet (upward arrow), (B) regurgitant jet across the mitral valve with color flow doppler (arrow), and (C) mobile mass adherent to the anterior mitral valve leaflet (arrow).

**Figure 3:**
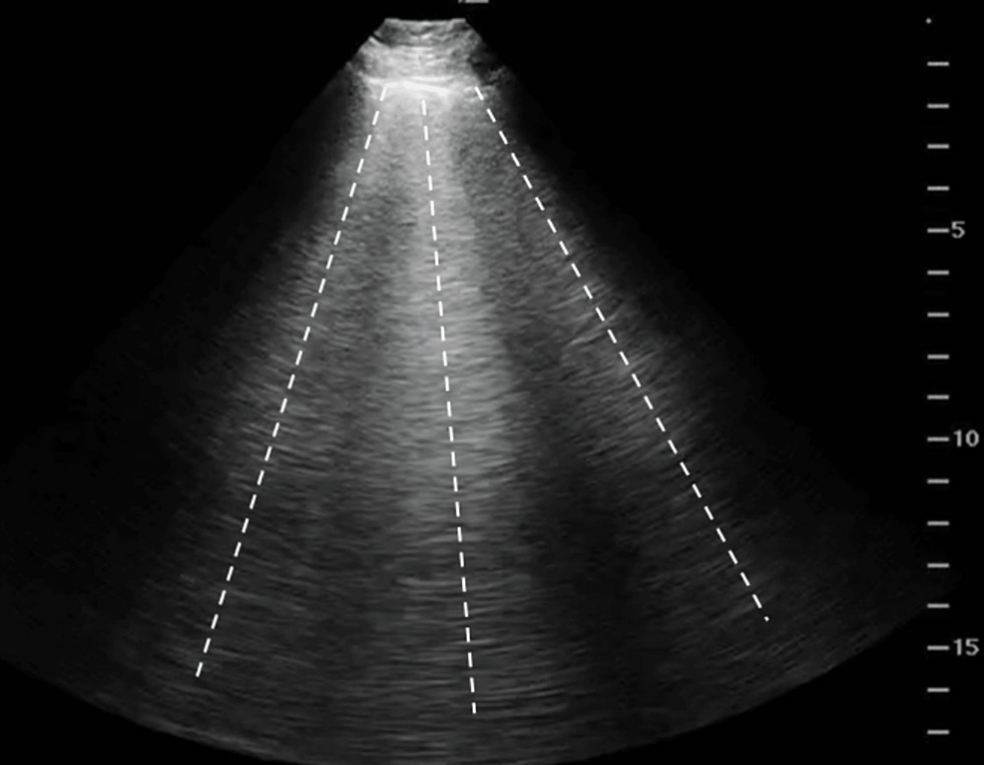
Point-of-care lung ultrasound Point-of-care lung ultrasound demonstrating diffuse b-lines (dotted lines) indicative of pulmonary edema.

**Figure 4:**
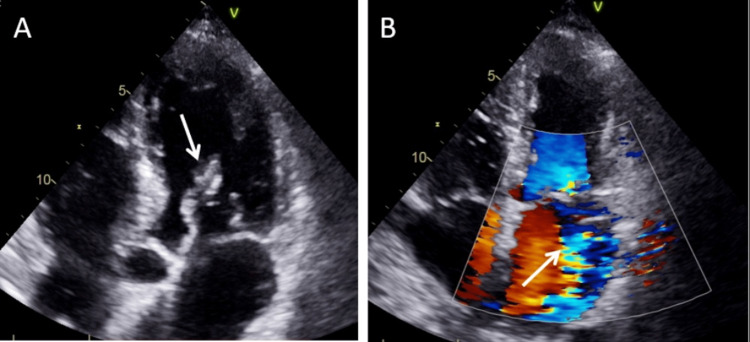
Comprehensive transthoracic echocardiogram Transthoracic echocardiogram (apical 5-chamber and 4-chamber views) performed by cardiac sonographer demonstrating (A) mass adherent to the anterior mitral valve leaflet (arrow) and (B) severe mitral valve regurgitation with color flow doppler (arrow).

While awaiting the arrival of the consulting cardiologist, the EP performed POCTE to estimate ejection fraction and assess volume status to guide further resuscitation. Unexpectedly, imaging revealed evidence of severe mitral valve regurgitation with the clear retrograde movement of mitral valve leaflets into the left atrium with a regurgitant jet across the mitral valve (Figure 2). Additional images obtained by the EP demonstrated a hyperdynamic left ventricle and pulmonary edema (Figure 3). An emergent comprehensive transthoracic echocardiogram confirmed these findings and further characterized a ruptured anterolateral papillary muscle with flail motion of the anterior mitral valve leaflet (Figure 4). In the interim, initial high-sensitivity troponin resulted at 3,835 ng/L (reference range <22 ng/L). The patient received emergent left heart catheterization with an initial diagnosis of late-presenting myocardial infarction complicated by cardiogenic shock from papillary muscle rupture and severe mitral valve regurgitation.

**Figure 5:**
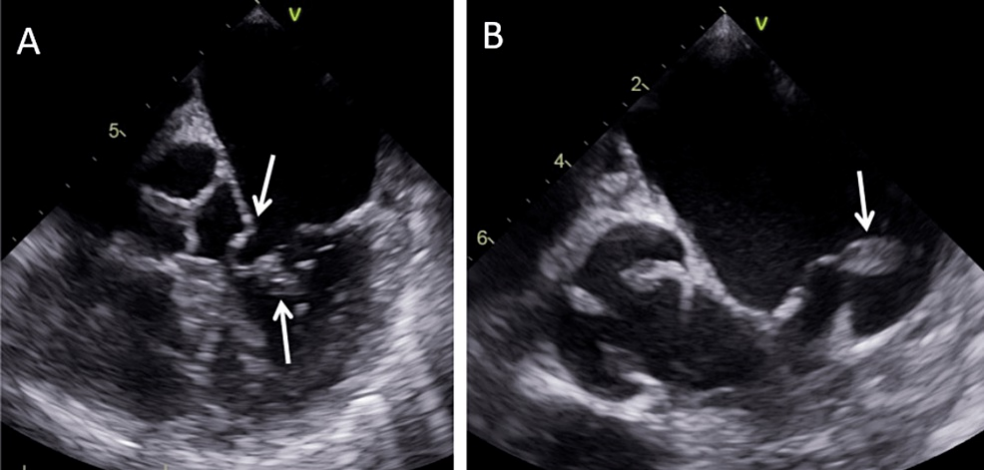
Comprehensive transesophageal echocardiogram Transesophageal echocardiogram (mid-esophageal 4-chamber view) demonstrating (A) flail motion of the anterior mitral valve leaflet (downward arrow) with adherent mass (upward arrow) and (B) ruptured anterolateral papillary muscle adherent to anterior mitral valve leaflet (arrow).

After endotracheal intubation and placement of an intra-aortic balloon pump, invasive coronary angiography revealed a 95% embolic-type lesion at the bifurcation of the left anterior descending artery and first diagonal artery. Otherwise, the patient had little to no underlying coronary artery disease (isolated 40% tubular stenosis of the proximal left circumflex artery), normal left ventricular function (ejection fraction 60-65%), no wall motion abnormalities, and elevated biventricular filling pressures (right atrial pressure 16 mmHg; pulmonary artery pressure 43/22 mmHg; pulmonary capillary wedge pressure 25 mmHg with v waves up to 40 mmHg). Comprehensive TEE confirmed anterolateral papillary muscle rupture with severe mitral valve regurgitation, moderate aortic insufficiency, and normal ejection fraction (65%), but also identified a mass adherent to the anterior mitral valve leaflet (Figure 5) consistent with endocarditis. In retrospect, the valvular mass is also visible on both point-of-care and comprehensive transthoracic echocardiograms (Figures 2-4), but this was not appreciated by either ED or cardiology clinical staff until TEE in the cardiac catheterization suite.

The patient remained intubated and was transferred to the cardiothoracic intensive care unit on mechanical circulatory support (balloon pump), multiple vasopressors, vancomycin, and cefepime. Additional history from family members was notable for symptoms of generalized illness, fatigue, and malaise for several weeks prior to presentation, in addition to endocarditis risk factors of poor dentition and ethanol use disorder. His cardiogenic shock steadily worsened over the subsequent 24 hours with progressive renal failure, lactic acidosis, and escalating vasopressor and inotropic requirements. His family declined surgical intervention and initiation of hemodialysis, and the patient transitioned to comfort care and expired shortly thereafter on hospital day two. Initial blood cultures were negative, and his family declined an autopsy. Ultimately, the papillary muscle rupture was attributed to endocarditis over embolic occlusion of the first diagonal artery.

## Discussion

POCTE is an important tool for the EP evaluating a patient in shock [5]. Acute severe mitral valve regurgitation is an etiology of shock that can be difficult to diagnose clinically but is identifiable on POCTE in the ED. Sonographic features include a regurgitant jet from the left ventricle to the left atrium with color doppler, hyperdynamic left ventricle, papillary muscle head visualized in the left atrium, dilated inferior vena cava with minimal respiratory variation, pleural effusions, and pulmonary edema [3,4].

Etiologies of papillary muscle rupture leading to acute mitral valve regurgitation include myocardial ischemia, endocarditis, myocarditis, trauma, Takotsubo syndrome, and connective tissue disorders [6]. In cases of endocarditis, acute mitral valve regurgitation is most commonly due to the direct destruction of the valve itself, whereas papillary muscle rupture is largely described in case reports [7,8]. Papillary muscle rupture in the context of endocarditis can result from coronary artery embolism with ischemic necrosis, regurgitation of a seeded aortic valve with bacterial deposition, or direct invasion by the infectious organism [7,8].

Acute coronary syndrome (ACS) complicates 2-3% of cases of infectious endocarditis and can result from septic embolization, external compression of a coronary artery by abscess or pseudoaneurysm, mechanical obstruction of a coronary artery by the vegetation, severe aortic regurgitation with a coronary steal, or inflammatory coronary thrombosis superimposed on pre-existing stenosis [9,10]. Patients with endocarditis complicated by ACS have a significantly higher risk of acute heart failure, cardiogenic shock, and death [9,10]. It is prudent to identify endocarditis as the etiology of ACS since traditional percutaneous interventions for ACS risk iatrogenic complications when infectious emboli are present. Balloon angioplasty risks additional embolism, stent implantation risks coronary artery mycotic aneurysm or stent infection, and thrombolytics carry a significantly higher risk of hemorrhagic complications [11,12]. Often, a multi-disciplinary team of cardiology, cardiothoracic surgery, and critical care navigates treatment options for these patients. Although this patient’s initial set of blood cultures was negative and his family declined an autopsy, endocarditis remains the most likely unifying diagnosis, given the combination of mitral valve vegetation, papillary muscle rupture, embolic coronary occlusion, and normal left ventricular function. Blood culture-negative endocarditis (BCNE) is a well-described entity that occurs when blood cultures are sterilized by prior antibacterial treatment, a fastidious microorganism requires prolonged incubation to isolate, or intracellular bacteria cannot be cultured in blood with available laboratory methods [13]. Additional testing to identify causative microorganisms in BCNE includes systematized testing of blood and/or valvular biopsy specimens using serological, molecular, and histopathological assays [13].

In this case, POCTE by the EP rapidly identified the presence of severe mitral valve regurgitation contributing to the clinical picture of STEMI with cardiogenic shock. In retrospect, the valvular vegetation is visible on both point-of-care and comprehensive echocardiography but was not recognized by the ED senior resident, the ED attending, the cardiology physician assistant, or the cardiology fellow until subsequent coronary angiography and comprehensive TEE in the cardiac catheterization suite. The patient’s critical illness and initial diagnosis appropriately prompted this TEE within one hour of POCTE in the ED, at which point the diagnosis was revised to endocarditis complicated by embolic coronary occlusion and papillary muscle rupture with severe mitral valve regurgitation. The diagnostic sequence in this case highlights several salient items. First, depending on the size of vegetation and underlying valvular disease, TEE is more sensitive (90-100%) than transthoracic echocardiography (40-63%) to identify infective endocarditis in native heart valves [14]. Second, point-of-care ultrasound (PoCUS) is designed to address specific clinical questions in specific clinical contexts [15]. When used to assess for evidence of endocarditis in a high-risk population, POCTE has reasonable diagnostic test accuracy. In a multi-center, cross-sectional study of 258 adults with bacteremia or candidemia, POCTE performed by internists had 77% sensitivity and 94% specificity to detect valvular vegetations against the reference standard of TEE performed by cardiologists [16]. However, in this patient, the preceding symptoms of malaise and generalized illness were not fully characterized until admission to the intensive care unit, and endocarditis was not otherwise entertained upon initial presentation to the ED with STEMI and cardiogenic shock. Ostensibly, the ED and consulting cardiology clinical staff would have been more likely to recognize the mitral valve mass if its identification had been the focus of the POCTE assessment. Thus, conflating PoCUS as a broad screening tool risks premature diagnostic closure if either PoCUS is insufficiently sensitive to detect relevant pathology or the relevant pathology is not recognized by the interpreting clinician. This case reinforces that PoCUS addresses a discrete clinical question and cautions clinicians against over-interpreting PoCUS to be ‘otherwise normal’ beyond the scope of its focused assessment. POCTE in the ED is typically directed at identifying pericardial effusion or tamponade, identifying cardiac activity, global assessing contractility, and characterizing central venous volume status [15]. Identification of valvular insufficiency and/or vegetation is not typically included but is feasible by an experienced sonographer [3,4,14].

## Conclusions

In this unusual case of an elderly male with STEMI complicated by cardiogenic shock, POCTE by the EP identified severe mitral valve regurgitation but did not identify the associated mitral valve vegetation. After invasive coronary angiography and comprehensive TEE, the initial diagnosis of myocardial infarction complicated by papillary muscle rupture and severe mitral insufficiency was revised to endocarditis with secondary embolic coronary occlusion of the first diagonal artery, myocardial infarction, papillary muscle rupture, and severe mitral insufficiency. ACS occurs in only 2-3% of patients with endocarditis and papillary muscle rupture is an uncommon occurrence in endocarditis largely described in case reports. This case features simultaneous uncommon complications of endocarditis and cautions against over-interpretation of clinician-performed PoCUS assessment beyond its specific clinical focus.
